# Tripartite Quantum Steering Dynamics in Photonic Systems Under Non-Markovian Dynamics

**DOI:** 10.3390/e28060602

**Published:** 2026-05-27

**Authors:** Smail Bougouffa, Kamal Berrada

**Affiliations:** Department of Physics, College of Science, Imam Mohammad Ibn Saud Islamic University (IMSIU), Riyadh 11432, Saudi Arabia; kaberrada@imamu.edu.sa

**Keywords:** quantum steering, photonic networks, Fabry–Pérot cavity, interference filter, open quantum systems, tripartite correlation, 03.67.-a, 03.65.Yz, 03.65.Ud

## Abstract

We investigate the non-Markovian dynamics of quantum steering in a tripartite photonic system subject to dephasing noise. By developing a theoretical framework based on the single-photon dephasing model extended to three independent photons, we analyze the temporal evolution of steering measures $S^{A-BC}$ and $S^{AB-C}$ for two distinct classes of initial states: W-type entangled states and GHZ-type mixed entangled states. The system is studied under various environmental configurations, ranging from fully Markovian to fully non-Markovian regimes, with asymmetric distributions of memory effects across the three photons. Our results reveal that the dynamics of tripartite steering are highly sensitive to both the number of photons coupled to non-Markovian environments and the specific partition of the system being considered. For W-states, non-Markovian effects induce oscillatory behavior with death–revival cycles, where the intervals of sudden death and revival amplitudes depend critically on the distribution of memory effects. For GHZ-states, we observe multiple death–revival cycles in some configurations and prolonged preservation of steering without complete sudden death in others. Notably, we find that non-Markovian environments significantly influence the dynamics of quantum steering through information backflow effects, with their impact depending sensitively on the subsystem to which the environment is coupled and on the roles of the steering and steered parties. These findings demonstrate that non-Markovian effects can significantly influence the preservation and degradation of directional quantum correlations, with their impact depending strongly on the coupling configuration and the choice of steering and steered subsystems. This behavior provides useful insight into the control of quantum steering in photonic networks and related quantum information processing tasks.

## 1. Introduction

Quantum steering traces back to Schrödinger’s 1936 reply to the EPR paradox [1,2]. It refers to the ability of one party to direct the state of a distant quantum system using shared entanglement [3,4,5,6,7], and provides a way to confirm entanglement without relying on the measurement devices. In 2007 Wiseman and co-workers revived the idea by giving it a precise operational definition [8]. They placed steering between ordinary entanglement and Bell nonlocality: it is strictly stronger than entanglement but weaker than the full nonlocality revealed by Bell tests. This definition underlines the one-way nature of steering, which has since been observed in both continuous-variable and discrete-variable quantum systems [9,10,11,12,13,14,15]. The two-party case naturally extends to the multi-party setting, where more complex forms of correlation appear. In the three-party case, Reid’s monogamy relationship holds: a single party can steer both others at the same time, but two independent parties cannot steer the same third party [16,17]. Tripartite systems also display layered steering structures that include genuine three-party steering, effective two-party steering, and collective steering. These rich structures position multipartite steering as a key resource for designing and analyzing quantum networks [18]. Consequently, the special properties of multipartite steering support new protocols for large-scale quantum communication and establish steering itself as a useful quantum resource.

Controlling the temporal evolution of quantum steering and mitigating decoherence are essential for reliable quantum information processing. In photonic systems, environmental dephasing commonly induces the sudden death of steering [11,19,20], thereby restricting the operational range of quantum communication and networking protocols. Photonic platforms nevertheless provide important advantages over other implementations, including extended coherence times and compatibility with high-speed, long-distance optical channels, which make them especially suitable for scalable quantum networks [21,22]. However, photon loss, phase damping, and spectral diffusion continue to challenge the long-term preservation of directional quantum correlations.

Despite significant progress in understanding bipartite steering dynamics, the tripartite regime remains considerably less explored, particularly in the presence of environmental memory effects. In multipartite settings, the interplay between system partitioning, steering directionality, and noise correlations can lead to qualitatively different dynamical behaviors that are not captured by two-party analyses.

Quantum steering occupies a distinctive position among quantum resources: it is strictly stronger than entanglement yet weaker than Bell nonlocality and possesses an inherent one-way character that enables device-independent entanglement verification. Quantum steering plays a central role in scenarios where entanglement alone is insufficient to characterize directional quantum correlations. In particular, steering provides an operational framework for asymmetric quantum information tasks such as one-sided device-independent protocols and quantum network verification, where the trust in measurement devices is not symmetric between parties. Although the dynamics of entanglement and nonlocality under noise have received considerable attention, the behavior of steering—particularly in the tripartite regime—requires further investigation [9]. Non-Markovian environments, where memory effects permit the backflow of information from the reservoir to the system, can counteract irreversible decay and induce periodic revivals of quantum steering [23,24,25].

However, the role of non-Markovianity in multipartite steering is not yet fully understood, as memory effects may act either as a resource or as a source of enhanced decoherence depending on the system configuration and the distribution of environmental coupling. This ambiguity motivates a systematic study of steering dynamics under different memory distributions across subsystems.

To address these issues, we investigate the non-Markovian dynamics of quantum steering in a tripartite photonic system subject to dephasing noise. By developing a theoretical framework based on the single-photon dephasing model extended to three independent photons, we analyse the temporal evolution of the steering measures $S^{A\to BC}$ and $S^{AB\to C}$ for two distinct classes of initial states: W-type entangled states and GHZ-type mixed entangled states. The system is studied under various environmental configurations ranging from fully Markovian to fully non-Markovian regimes, with asymmetric distributions of memory effects across the three photons. Our results reveal that the dynamics of quantum steering are highly sensitive to both the number of photons coupled to non-Markovian environments and the specific partition of the system being considered. Non-Markovian environments can either enhance steering through information backflow or prove detrimental depending on which subsystems they are coupled to relative to the steering and steered parties. These results indicate that environmental memory effects can substantially modify the decay dynamics of multipartite steering by enabling partial recovery of coherence through information backflow mechanisms.

In particular, we show that the presence of non-Markovian memory does not guarantee improved robustness of steering correlations, and in certain configurations it may lead to faster decay than in the Markovian regime despite exhibiting revival behavior.

While previous studies have extensively investigated bipartite steering dynamics and the influence of non-Markovian environments on entanglement and nonlocality, comparatively less attention has been devoted to tripartite steering in discrete-variable photonic systems with asymmetric memory distributions. In particular, the combined influence of steering directionality, multipartite partitioning, and non-uniform distributions of Markovian and non-Markovian environments remains insufficiently explored. The present work addresses this gap by systematically analyzing the steering dynamics of W-type and GHZ-type three-photon states under several distinct memory configurations, thereby revealing how environmental memory effects depend not only on the reservoir properties but also on the specific steering partition considered.

The structure of this paper is as follows. In Section 2, we develop the theoretical framework for non-Markovian dynamics in photonic networks. We begin by introducing the single-photon dephasing model, which we then extend to describe three independent photons interacting with their respective environments. A matrix representation in the polarization basis is presented to accurately capture the dephasing mechanisms affecting each photon. In Section 3, we apply this framework to analyze the temporal evolution of W-type entangled states under both Markovian and non-Markovian dephasing dynamics. Section 4 extends this analysis to GHZ-type mixed entangled states, examining how environmental memory effects influence their decay and revival patterns. The concept of quantum steering in three-photon systems is introduced in Section 5, where we define the steering measures $S^{A-BC}$ and $S^{AB-C}$ used to quantify directional quantum correlations between different bipartitions. Our main findings are presented and discussed in Section 6, where we systematically compare the steering dynamics for both W and GHZ states under various environmental configurations, ranging from fully Markovian to fully non-Markovian regimes with asymmetric distributions of memory effects across the three photons. Finally, Section 7 summarizes our conclusions and discusses the implications of our results for quantum information processing tasks requiring the preservation of directional quantum correlations in photonic networks.

## 2. Non-Markovian Evolution in Structured Photonic Reservoirs

In this section we formulate a general theoretical framework describing memory-dependent dynamics in photonic systems. We begin by analyzing a single-photon polarization qubit interacting with a structured frequency reservoir, and then extend the description to a three-photon configuration. This progressive construction highlights how environmental memory effects become more intricate as the Hilbert-space dimension increases and provides a systematic approach for modeling decoherence in multi-photon quantum networks. We assume that each photon experiences pure dephasing induced by its coupling between polarization and frequency degrees of freedom, which leads to a loss of coherence without population transfer. This provides a minimal but physically realistic model for decoherence in optical quantum networks.

### 2.1. Single-Photon Dephasing Mechanism

**Figure 1:**
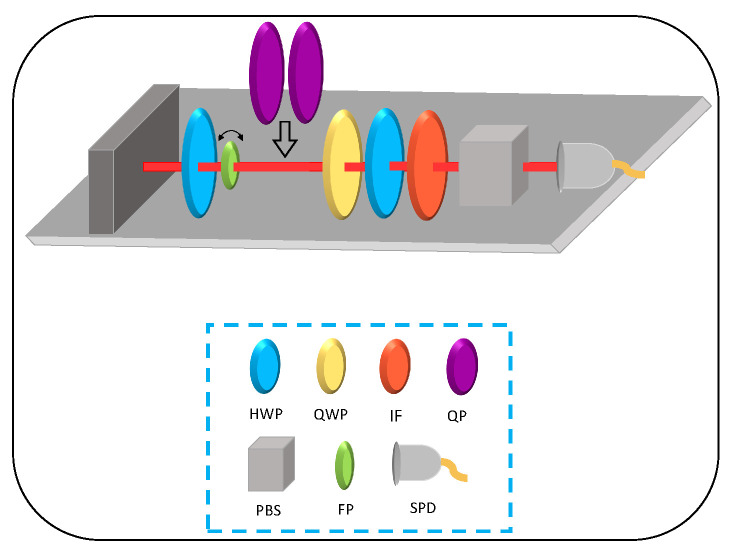
This figure provides a schematic illustration of the theoretical model examined in this work. It conceptually depicts the interaction between polarization and frequency in a standard optical configuration, thereby outlining the overall theoretical framework. The diagram features several labeled optical components—half-wave plate (HWP), quarter-wave plate (QWP), interference filter (IF), quartz plate (QP), polarizing beam splitter (PBS), Fabry–Pérot cavity (FP), and single-photon detector (SPD)—to demonstrate how these polarization and frequency degrees of freedom can, in principle, be controlled and manipulated.

Non-Markovianity in this setting arises from the structured frequency distribution of the photon, which introduces memory effects through the environmental spectral profile rather than through direct system–environment back-action. We consider an open quantum system where the system is encoded in the polarization of a photon, while the environmental degree of freedom is associated with its frequency. Experimentally, controllable non-Markovian dynamics can be realized using a tunable Fabry–Pérot (FP) cavity combined with an interference filter and a birefringent quartz plate [21,25,26,27,28]. The schematic illustration of the theoretical model is presented in Figure 1. The FP cavity generates a comb-like spectral distribution, while the interference filter isolates two dominant frequency components.

The normalized spectral probability density of the photon is denoted by g(ω) and is modeled as a weighted sum of two Gaussian functions [29]:

(1) $$g\left(\omega \right)=\frac{\cos^{2}\theta }{\sqrt{2\pi }\sigma }e^{-\frac{{\left(\omega -\omega_{1}\right)}^{2}}{2\sigma^{2}}}+\frac{\sin^{2}\theta }{\sqrt{2\pi }\sigma }e^{-\frac{{\left(\omega -\omega_{2}\right)}^{2}}{2\sigma^{2}}},$$

where $\omega_{1}$ and $\omega_{2}$ denote the central frequencies, σ is the common spectral width, and θ∈[0,π/2] determines the relative weight of the two spectral peaks. The tilt of the FP cavity effectively controls the value of θ, allowing one to tune the spectral asymmetry. The parameter θ therefore controls the degree of spectral imbalance, which in turn determines the strength of interference between the two frequency components and directly affects the non-Markovian character of the induced dephasing dynamics.

The non-Markovian behavior considered here originates from the structured spectral distribution of the environmental frequency modes. As the polarization and frequency degrees of freedom become correlated during the evolution, coherence information is temporarily transferred to the environmental sector and may subsequently flow back into the polarization subsystem through spectral interference effects. The resulting oscillatory revival behavior therefore reflects memory-induced information backflow associated with the finite spectral structure of the reservoir after tracing over the environmental frequency modes.

When the photon propagates through the birefringent quartz plate, its polarization components accumulate different frequency-dependent phases, resulting in a pure dephasing process. The interaction Hamiltonian describing the coupling between polarization and frequency degrees of freedom is given by [30]

(2) $$H_{SE}=-\int \left(n_{V}\left|V\rangle \langle V\right|+n_{H}\left|H\rangle \langle H\right|\right)⊗\omega |\omega \rangle \langle \omega |\;d\omega ,$$

where $n_{V}$ and $n_{H}$ are the refractive indices corresponding to vertical and horizontal polarizations, respectively. This interaction generates a relative phase shift between polarization components that depends on frequency. This interaction produces a polarization-dependent phase shift that depends continuously on the photon frequency, thereby generating pure dephasing in the polarization basis.

The initial system–environment state is assumed to be separable:

$$\rho_{SE}\left(0\right)=\rho_{S}\left(0\right)⊗\rho_{E}\left(0\right),$$

with the environmental density operator

(3) $$\rho_{E}\left(0\right)=\int d\omega \;d\omega^{\prime }\;G\left(\omega \right)G^{*}\left(\omega^{\prime }\right)|\omega \rangle \langle \omega^{\prime }|,\;|G\left(\omega \right){|}^{2}=g\left(\omega \right).$$

The reduced system dynamics is obtained through

(4) $$\rho_{S}\left(t\right)={\mathrm{Tr}}_{E}\;\left[U\left(t\right)\rho_{SE}\left(0\right)U^{†}\left(t\right)\right],\;U\left(t\right)=e^{-iH_{SE}t}.$$

In the polarization basis {|V〉,|H〉}, the evolved density matrix takes the form [31]

(5) $$\rho_{S}\left(t\right)=\left(\begin{array}{cc} \rho_{VV}\left(0\right) & \rho_{VH}\left(0\right)\eta \left(t\right)\\ \rho_{HV}\left(0\right)\eta^{*}\left(t\right) & \rho_{HH}\left(0\right) \end{array}\right),$$

where the dephasing function is

(6) $$\eta \left(t\right)=\int g\left(\omega \right)\;e^{i\omega \Delta nt}\;d\omega ,\;\Delta n=n_{V}-n_{H}.$$

Substituting Equation (1) into Equation (6) yields

(7) $$\eta \left(t\right)=e^{-\frac{1}{2}\sigma^{2}{\left(\Delta nt\right)}^{2}}\left(e^{i\omega_{1}\Delta nt}\cos^{2}\theta +e^{i\omega_{2}\Delta nt}\sin^{2}\theta \right).$$

This single-photon model provides the building block for constructing multipartite dynamics, where each subsystem evolves independently under an identical but locally applied dephasing mechanism.

### 2.2. Generalization to Three Independent Photons

We now consider three non-interacting polarization qubits labeled *A*, *B*, and *C*, each associated with an individual photon. Each subsystem interacts locally with its own structured environment $E_{A}$, $E_{B}$, and $E_{C}$. The resulting dynamics can be equivalently described using a Kraus representation [32], which allows us to express the non-unitary evolution in a completely positive and trace-preserving form.

The reduced dynamics of subsystem S∈{A,B,C} can be expressed using a completely positive trace-preserving map:

(8) $$\rho_{S}\left(t\right)=\sum_{m,n}K_{mn}^{\left(S\right)}\left(t\right)\;\rho_{S}\left(0\right)\;K_{mn}^{(S)†}\left(t\right),$$

where the Kraus operators encode local decoherence characterized by the function $\eta_{S}\left(t\right)$ determined by the local spectral distribution [32].

Since the photons do not interact directly, the total evolution operator factorizes as

(9) $$U_{ABC}\left(t\right)=U_{A}\left(t\right)⊗U_{B}\left(t\right)⊗U_{C}\left(t\right).$$

The global density matrix evolves according to

(10) $$\rho_{ABC}\left(t\right)=\sum_{m,n,p}K_{m}^{\left(A\right)}\left(t\right)⊗K_{n}^{\left(B\right)}\left(t\right)⊗K_{p}^{\left(C\right)}\left(t\right)\;\rho_{ABC}\left(0\right)\;{\left(K_{m}^{\left(A\right)}⊗K_{n}^{\left(B\right)}⊗K_{p}^{\left(C\right)}\right)}^{†}.$$

This factorization reflects the assumption of independent reservoirs with no cross-correlations between photons, which is a standard approximation in photonic dephasing models.

### 2.3. Matrix Representation in the Polarization Basis

Using the local basis {|V〉,|H〉}, the reduced dynamics of each subsystem can be written as

(11) $$\rho_{\mu^{\prime }\nu^{\prime }}^{\left(S\right)}\left(t\right)=\sum_{\mu ,\nu }T_{\mu^{\prime }\nu^{\prime };\mu \nu }^{\left(S\right)}\left(t\right)\rho_{\mu \nu }^{\left(S\right)}\left(0\right),$$

where

(12) $$T_{\mu^{\prime }\nu^{\prime };\mu \nu }^{\left(S\right)}\left(t\right)=\sum_{m,n}\langle \mu^{\prime }|K_{mn}^{\left(S\right)}\left|\mu \rangle \langle \nu \right|K_{mn}^{(S)†}|\nu^{\prime }\rangle .$$

The tripartite Hilbert space is spanned by

(13) $$\begin{array}{c} B\;=\;\{|V_{A}V_{B}V_{C}\rangle ,\;|V_{A}V_{B}H_{C}\rangle ,\;|V_{A}H_{B}V_{C}\rangle ,\;|V_{A}H_{B}H_{C}\rangle ,\\ \;|H_{A}V_{B}V_{C}\rangle ,\;|H_{A}V_{B}H_{C}\rangle ,\;|H_{A}H_{B}V_{C}\rangle ,\;|H_{A}H_{B}H_{C}\rangle \}. \end{array}$$

Under local dephasing, populations remain invariant while coherences decay according to products of single-photon decoherence functions, reflecting the absence of energy exchange and the purely phase-damping nature of the environment.

(14) $$\rho_{ii}\left(t\right)=\rho_{ii}\left(0\right),\;i=1,\ldots ,7,\;\rho_{88}\left(t\right)=1-\sum_{i=1}^{7}\rho_{ii}\left(0\right).$$

The coherence terms evolve according to products of local decoherence functions:

(15) $$\begin{array}{ccc} \rho_{12}\left(t\right)=\rho_{12}\left(0\right)\eta_{C}\left(t\right), & \rho_{13}\left(t\right)=\rho_{13}\left(0\right)\eta_{B}\left(t\right), & \rho_{15}\left(t\right)=\rho_{15}\left(0\right)\eta_{A}\left(t\right),\\ \rho_{24}\left(t\right)=\rho_{24}\left(0\right)\eta_{B}\left(t\right), & \rho_{26}\left(t\right)=\rho_{26}\left(0\right)\eta_{A}\left(t\right), & \rho_{37}\left(t\right)=\rho_{37}\left(0\right)\eta_{A}\left(t\right),\\ \rho_{34}\left(t\right)=\rho_{34}\left(0\right)\eta_{C}\left(t\right), & \rho_{48}\left(t\right)=\rho_{48}\left(0\right)\eta_{A}\left(t\right), & \rho_{56}\left(t\right)=\rho_{56}\left(0\right)\eta_{C}\left(t\right),\\ \rho_{57}\left(t\right)=\rho_{57}\left(0\right)\eta_{B}\left(t\right), & \rho_{68}\left(t\right)=\rho_{68}\left(0\right)\eta_{B}\left(t\right), & \rho_{78}\left(t\right)=\rho_{78}\left(0\right)\eta_{C}\left(t\right),\\ \rho_{14}\left(t\right)=\rho_{14}\left(0\right)\eta_{B}\left(t\right)\eta_{C}\left(t\right), & \rho_{16}\left(t\right)=\rho_{16}\left(0\right)\eta_{A}\left(t\right)\eta_{C}\left(t\right), & \\ \rho_{17}\left(t\right)=\rho_{17}\left(0\right)\eta_{A}\left(t\right)\eta_{B}\left(t\right), & \rho_{23}\left(t\right)=\rho_{23}\left(0\right)\eta_{B}\left(t\right)\eta_{C}^{*}\left(t\right), & \\ \rho_{25}\left(t\right)=\rho_{25}\left(0\right)\eta_{A}\left(t\right)\eta_{C}^{*}\left(t\right), & \rho_{28}\left(t\right)=\rho_{28}\left(0\right)\eta_{A}\left(t\right)\eta_{B}\left(t\right), & \\ \rho_{35}\left(t\right)=\rho_{35}\left(0\right)\eta_{A}\left(t\right)\eta_{B}^{*}\left(t\right), & \rho_{38}\left(t\right)=\rho_{38}\left(0\right)\eta_{A}\left(t\right)\eta_{C}\left(t\right), & \\ \rho_{46}\left(t\right)=\rho_{46}\left(0\right)\eta_{A}\left(t\right)\eta_{B}^{*}\left(t\right), & \rho_{47}\left(t\right)=\rho_{47}\left(0\right)\eta_{A}\left(t\right)\eta_{C}^{*}\left(t\right), & \\ \rho_{58}\left(t\right)=\rho_{58}\left(0\right)\eta_{B}\left(t\right)\eta_{C}\left(t\right), & \rho_{67}\left(t\right)=\rho_{67}\left(0\right)\eta_{B}\left(t\right)\eta_{C}^{*}\left(t\right), & \\ \rho_{18}\left(t\right)=\rho_{18}\left(0\right)\eta_{A}\left(t\right)\eta_{B}\left(t\right)\eta_{C}\left(t\right), & \rho_{27}\left(t\right)=\rho_{27}\left(0\right)\eta_{A}\left(t\right)\eta_{B}\left(t\right)\eta_{C}^{*}\left(t\right), & \\ \rho_{36}\left(t\right)=\rho_{36}\left(0\right)\eta_{A}\left(t\right)\eta_{B}^{*}\left(t\right)\eta_{C}\left(t\right),\;\;\;\;\;\;\;\;\; & \rho_{45}\left(t\right)=\rho_{45}\left(0\right)\eta_{A}\left(t\right)\eta_{B}^{*}\left(t\right)\eta_{C}^{*}\left(t\right). & \end{array}$$

Hermiticity is preserved automatically, ensuring $\rho_{ABC}\left(t\right)=\rho_{ABC}^{†}\left(t\right)$.

Equations (14) and (15) provide a complete description of the three-qubit density matrix evolution under local non-Markovian dephasing, where collective coherence decay is governed by products of individual decoherence functions. This formalism provides a transparent description of how local memory effects combine multiplicatively to shape the global decay and revival of multipartite coherences, thereby enabling a systematic study of steering dynamics in structured photonic environments.

## 3. Temporal Evolution of W-Type Entangled State

This section examines how a W-type state changes over time in a system made up of three qubits.

We consider the three-qubit W-state as the initial resource:

(16) $$|\Psi_{W}\rangle =\frac{1}{\sqrt{3}}\left(|H_{A}V_{B}V_{C}\rangle +|V_{A}H_{B}V_{C}\rangle +|V_{A}V_{B}H_{C}\rangle \right),$$

which is a paradigmatic example of a tripartite entangled state. W-states are distinguished by their robustness: if one qubit is lost, the remaining two qubits retain some entanglement, making them particularly suitable for noisy quantum systems and distributed quantum protocols [33]. They can be generated experimentally [34] using sequences of quantum gates or optical setups, and they play a central role in quantum secret sharing, quantum networks, and error-correcting schemes.

The initial density matrix is defined as

(17) $$\rho (0)=|\Psi_{W}\rangle \langle \Psi_{W}|.$$

In the standard three-qubit basis (13), the density matrix takes the explicit 8×8 form

(18) $$\rho \left(0\right)=\frac{1}{3}\left(\begin{array}{cccccccc} 0 & 0 & 0 & 0 & 0 & 0 & 0 & 0\\ 0 & 1 & 1 & 0 & 1 & 0 & 0 & 0\\ 0 & 1 & 1 & 0 & 1 & 0 & 0 & 0\\ 0 & 0 & 0 & 0 & 0 & 0 & 0 & 0\\ 0 & 1 & 1 & 0 & 1 & 0 & 0 & 0\\ 0 & 0 & 0 & 0 & 0 & 0 & 0 & 0\\ 0 & 0 & 0 & 0 & 0 & 0 & 0 & 0\\ 0 & 0 & 0 & 0 & 0 & 0 & 0 & 0 \end{array}\right).$$

This matrix represents the W-state in the full three-qubit Hilbert space, with nonzero entries only in the block 3×3 corresponding to the states $|H_{A}V_{B}V_{C}\rangle$, $|V_{A}H_{B}V_{C}\rangle$, and $|V_{A}V_{B}H_{C}\rangle$. The time evolution of the density matrix under decoherence can be obtained using the general formalism of Equations (14) and (15). The corresponding time-dependent density matrix is

(19) $$\rho \left(t\right)=\frac{1}{3}\left(\begin{array}{cccccccc} 0 & 0 & 0 & 0 & 0 & 0 & 0 & 0\\ 0 & 1 & \eta_{B}\left(t\right)\eta_{C}^{*}\left(t\right) & 0 & \eta_{A}\left(t\right)\eta_{C}^{*}\left(t\right) & 0 & 0 & 0\\ 0 & \eta_{B}^{*}\left(t\right)\eta_{C}\left(t\right) & 1 & 0 & \eta_{A}\left(t\right)\eta_{B}^{*}\left(t\right) & 0 & 0 & 0\\ 0 & 0 & 0 & 0 & 0 & 0 & 0 & 0\\ 0 & \eta_{A}^{*}\left(t\right)\eta_{C}\left(t\right) & \eta_{A}^{*}\left(t\right)\eta_{B}\left(t\right) & 0 & 1 & 0 & 0 & 0\\ 0 & 0 & 0 & 0 & 0 & 0 & 0 & 0\\ 0 & 0 & 0 & 0 & 0 & 0 & 0 & 0\\ 0 & 0 & 0 & 0 & 0 & 0 & 0 & 0 \end{array}\right).$$

This explicit representation allows for a straightforward analysis of the temporal decay of W-type entanglement and provides a clear basis for calculating multipartite entanglement measures.

## 4. Temporal Evolution of GHZ-Type Mixed Entangled State

This section explores the temporal evolution of a GHZ-type state in a system composed of three qubits. We consider a GHZ–type entangled configuration as the initial resource of the system. The GHZ state represents a fundamental class of tripartite entanglement distinct from the W-type family, exhibiting maximal nonlocal correlations and perfect coherence between the logical states $|V_{A}V_{B}V_{C}\rangle$ and $|H_{A}H_{B}H_{C}\rangle$. It is defined as

(20) $$|\mathrm{GHZ}\rangle =\frac{1}{\sqrt{2}}\left(|V_{A}V_{B}V_{C}\rangle +|H_{A}H_{B}H_{C}\rangle \right).$$

To account for realistic imperfections and partial mixing with environmental noise, we describe the initial state using a convex combination of a maximally mixed state and the pure GHZ state:

(21) $$\rho \left(0\right)=\frac{1-a}{8}\;I_{8}+a\;|\mathrm{GHZ}\rangle \langle \mathrm{GHZ}|,\;0\leq a\leq 1,$$

where *a* denotes the degree of purity or coherence. This mixed GHZ-type density operator serves as a realistic model for partially decohered entangled sources in photonic or superconducting qubit platforms. The GHZ state plays a central role in foundational studies of quantum nonlocality and multipartite correlations. Unlike W-type states, GHZ-type entanglement exhibits stronger violation of local realism and is particularly useful in testing Bell-type inequalities, quantum secret sharing, distributed quantum computation, and error-resistant quantum communication protocols. Its preparation and manipulation have been demonstrated in several physical systems, including trapped ions, photons, and superconducting circuits [35,36,37,38,39,40,41]. In contrast to the W-state, which retains partial bipartite entanglement after the loss of one qubit, the GHZ state is highly sensitive to particle loss—its entanglement vanishes if any qubit is traced out. However, this fragility is compensated by its stronger correlations and nonlocal features, making it a valuable testbed for studying decoherence mechanisms and the transition from quantum to classical behavior.

In the standard computational polarization basis (13), the initial state (21), which represents a convex combination of the maximally mixed state and the pure GHZ state. The parameter *a* quantifies the degree of purity, ranging from a completely mixed state (a=0) to the pure GHZ state (a=1). The pure component |GHZ〉〈GHZ| occupies a two-dimensional subspace spanned by the first and last computational basis vectors, while all other elements of the density matrix vanish. Consequently, in this basis, the initial density matrix takes the following form

(22) $$\rho \left(0\right)=\left(\begin{array}{cccccccc} c & 0 & 0 & 0 & 0 & 0 & 0 & \frac{a}{2}\\ 0 & d & 0 & 0 & 0 & 0 & 0 & 0\\ 0 & 0 & d & 0 & 0 & 0 & 0 & 0\\ 0 & 0 & 0 & d & 0 & 0 & 0 & 0\\ 0 & 0 & 0 & 0 & d & 0 & 0 & 0\\ 0 & 0 & 0 & 0 & 0 & d & 0 & 0\\ 0 & 0 & 0 & 0 & 0 & 0 & d & 0\\ \frac{a}{2} & 0 & 0 & 0 & 0 & 0 & 0 & c \end{array}\right),$$

where $c=\frac{1+3a}{8}$ and $d=\frac{1-a}{8}$. This form clearly identifies the coherent GHZ correlations through the off-diagonal terms $\rho_{18}=\rho_{81}=a/2$, which encode the superposition between the all-|V〉 and all-|H〉 components. As the system evolves under local decoherence channels, these terms decay, providing a quantitative signature of entanglement degradation and non-Markovian memory effects. By employing the relations established in Equations (14) and (15), the time-dependent density operator of the tripartite system, denoted by ρ(t), can be explicitly constructed. Since dephasing processes preserve the population terms, the diagonal elements of ρ(t) remain constant in time, while the coherence elements decay according to the product of the corresponding local decoherence functions $\eta_{S}\left(t\right)$ associated with each qubit subsystem S=A,B,C. Accordingly, the temporal form of the density matrix is expressed as

(23) $$\rho \left(t\right)=\left(\begin{array}{cccccccc} c & 0 & 0 & 0 & 0 & 0 & 0 & \frac{a}{2}\;\eta_{A}\left(t\right)\eta_{B}\left(t\right)\eta_{C}\left(t\right)\\ 0 & d & 0 & 0 & 0 & 0 & 0 & 0\\ 0 & 0 & d & 0 & 0 & 0 & 0 & 0\\ 0 & 0 & 0 & d & 0 & 0 & 0 & 0\\ 0 & 0 & 0 & 0 & d & 0 & 0 & 0\\ 0 & 0 & 0 & 0 & 0 & d & 0 & 0\\ 0 & 0 & 0 & 0 & 0 & 0 & d & 0\\ \frac{a}{2}\;\eta_{A}^{*}\left(t\right)\eta_{B}^{*}\left(t\right)\eta_{C}^{*}\left(t\right) & 0 & 0 & 0 & 0 & 0 & 0 & c \end{array}\right).$$

This explicit representation allows for a straightforward analysis of the temporal decay of GHZ-type entanglement and provides a clear basis for calculating multipartite entanglement measures in the photonic system.

## 5. Quantum Steering in Three-Photon System

**Figure 2:**
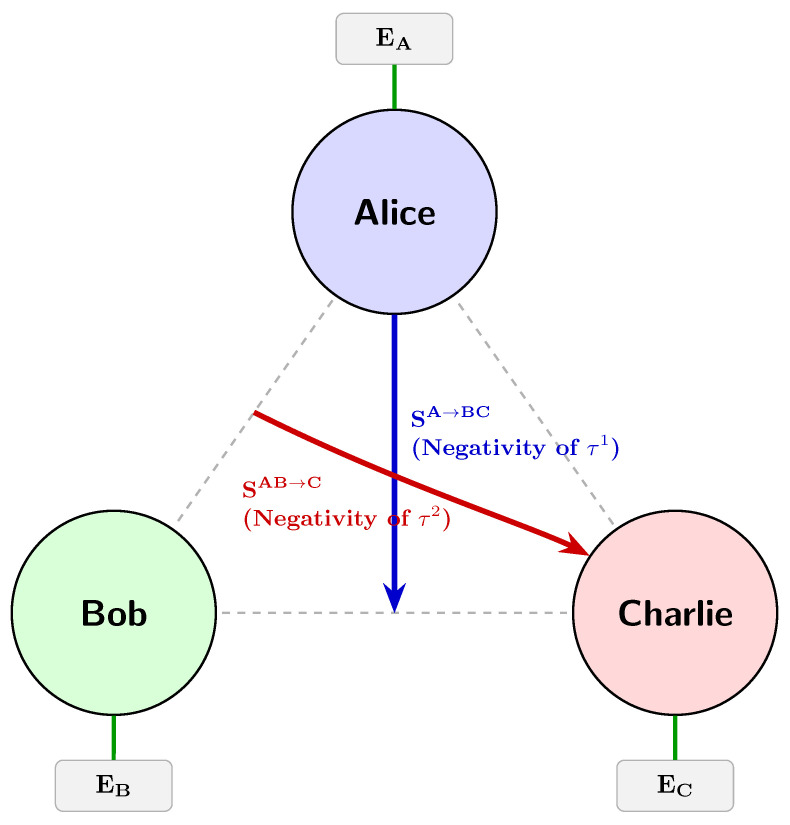
Schematic representation of the tripartite photonic system considered in this work. Three polarization qubits *A*, *B*, and *C*, prepared in either W-type or GHZ-type entangled states, interact locally with independent dephasing environments $E_{A}$, $E_{B}$, and $E_{C}$. The environmental couplings may correspond to either Markovian or non-Markovian regimes. The directional arrows indicate the steering configurations analyzed in this work: one-party steering $S^{A\to BC}$ and collective two-party steering $S^{AB\to C}$. The steering properties are quantified through the negativity of the corresponding matrices $\tau^{1}$ and $\tau^{2}$ introduced in Section 5.

The concept of tripartite quantum steering in the photonic system is illustrated in Figure 2. Three independent photons *A*, *B*, and *C* are prepared in either W-type or GHZ-type entangled states. Each photon couples locally to its own dephasing environment $E_{A}$, $E_{B}$, or $E_{C}$ (Markovian or non-Markovian). The steering properties are analyzed through two bipartitions: one-party steering $S^{A\to BC}$ and two-party steering $S^{AB\to C}$, quantified by the matrices $\tau^{1}$ and $\tau^{2}$, respectively. Steering is certified by the presence of negative eigenvalues of these matrices (see Appendix A for explicit forms).

The steering criterion used in this work is based on entanglement detection via the negativity of the partially transposed density operator. For completeness, we introduce the standard definition of the one-tangle as

(24) $$N_{A(BC)}=∥\rho_{ABC}^{T_{A}}∥-1,$$

where $T_{A}$ denotes partial transposition with respect to subsystem *A* [42]. This quantity is equivalent to twice the sum of the absolute values of negative eigenvalues of $\rho_{ABC}^{T_{A}}$:

(25) $$N_{A(BC)}=2\sum_{i=1}^{n}{\left|\lambda_{A(BC)}^{\left(-\right)}\right|}_{i},$$

where $|\lambda_{A(BC)}^{\left(-\right)}{|}_{i}$ are the negative eigenvalues of the partially transposed matrix.

For tripartite steering, we construct the witness matrices $\tau^{1}$ and $\tau^{2}$, whose explicit forms for W and GHZ states are given in Appendix A. A tripartite state $\rho_{ABC}$ is considered steerable in a given partition if the corresponding matrix $\tau^{1}$ or $\tau^{2}$ exhibits at least one negative eigenvalue.

The steering from Alice to Bob and Charlie can be witnessed if the density matrix $\tau_{ABC}^{1}$, defined as

(26) $$\tau_{ABC}^{1}=\frac{1}{\sqrt{3}}\rho_{ABC}+\alpha I_{2}⊗\rho_{BC},$$

is entangled, where $\rho_{BC}={\mathrm{Tr}}_{A}\left(\rho_{ABC}\right)$ and $I_{2}$ is the 2×2 identity matrix and $\alpha =\frac{\sqrt{3}-1}{2\sqrt{3}}$ [43].

Similarly, the steering from Alice and Bob to Charlie can be witnessed if the following density matrix is followed.

(27) $$\tau_{ABC}^{2}=\frac{1}{3}\rho_{ABC}+\frac{1}{6}I_{4}⊗\rho_{C},$$

is entangled, where $\rho_{C}={\mathrm{Tr}}_{AB}\left(\rho_{ABC}\right)$ and $I_{4}$ is the identity matrix 4×4 [43].

The steering criteria are determined from the eigenvalue spectra of the matrices $\tau^{1}$ and $\tau^{2}$. In particular, the appearance of at least one negative eigenvalue signals the violation of the corresponding steering inequality and therefore certifies the existence of steering for the associated bipartition.

## 6. Results and Discussion

**Figure 3:**
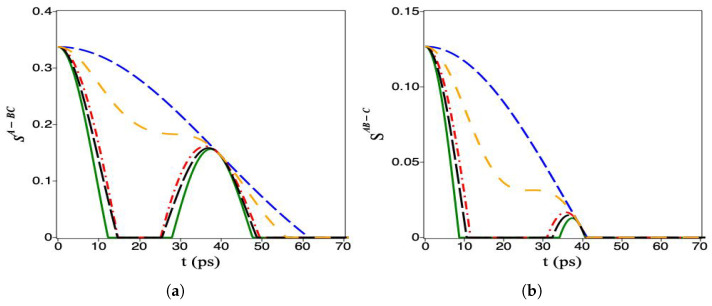
Time evolution of the steering measures $S^{A\to BC}$ (**a**) and $S^{AB\to C}$ (**b**) for a tripartite photonic W-state under Markovian and non-Markovian dephasing dynamics. The steering dynamics are shown for different distributions of environmental memory effects characterized by $\left(\theta_{A},\theta_{B},\theta_{C}\right)$. The system parameters are Δn=0.01, $\nu_{1}=2.676$ PHz, $\nu_{2}=2.692$ PHz, and σ=1.8 THz. Line colors denote different configurations: blue—fully Markovian case (0,0,0); green—fully non-Markovian case (π/4,π/4,π/4); red—single-photon non-Markovian case (π/4,0,0); black—two-photon non-Markovian case (π/4,π/4,0); orange—asymmetric case (0,π/4,0).

The dynamics of steering correlations for a three-mode photonic system, initially prepared in a W-state and subject to dephasing, are presented in Figure 3. The figure illustrates the time evolution of two distinct steering measures: $S^{A-BC}$ (panel a), which quantifies the ability of party *A* to steer the composite subsystem BC, and $S^{AB-C}$ (panel b), which quantifies the ability of the composite system AB to steer party *C*. The system evolves under various configurations of Markovian and non-Markovian dephasing environments, characterized by the angle parameter θ, with the spectral properties of the environments defined by the detuning Δn=0.01, central frequencies $\nu_{1}=2.676$ PHz and $\nu_{2}=2.692$ PHz, and bandwidth σ=1.8 THz [21]. The numerical parameters employed throughout this work are chosen in accordance with experimentally accessible values reported in Ref. [21], thereby maintaining a direct connection with realistic photonic implementations. Our intention is not to reproduce a specific experiment quantitatively, but rather to illustrate the qualitative features of multipartite steering dynamics under physically relevant conditions. We have verified that the main phenomena discussed in this work, including oscillatory decay, revival behavior, and the configuration-dependent influence of non-Markovianity, remain qualitatively robust under reasonable variations of the frequency and time-scale parameters.

We consider symmetric and asymmetric distributions of non-Markovianity characterized by different values of $(\theta_{A}$, $\theta_{B}$, $\theta_{C})$ corresponding to one-, two-, and three-photon memory configurations, respectively.

Panel (a) depicts the steering from mode *A* to the combined modes *B* and *C*. A key observation is the pronounced difference in the longevity of steering under different environmental conditions. In the fully Markovian symmetric case (dashed blue line, $\theta_{A}=\theta_{B}=\theta_{C}=0$), the steering decays monotonically and slowly, vanishing entirely by approximately t≈60 ps. This behavior is characteristic of memoryless environments, where information is lost irreversibly. In stark contrast, the fully non-Markovian symmetric case (solid green line, $\theta_{A}=\theta_{B}=\theta_{C}=\pi /4$) exhibits a rapid initial decay leading to a sudden death of steering. After a finite interval, however, the steering experiences a revival, displaying oscillatory behavior: it passes through a maximum before decaying once more to another sudden death. This oscillatory behavior is a direct consequence of the memory effects arising from the non-Markovian environments, which facilitate a temporary backflow of information between the tripartite system and its reservoirs, leading to the recurrent revival and decay of quantum correlations. The intermediate cases reveal a nuanced dependence on how many photons are coupled to a non-Markovian reservoir. The scenario with a single photon in a non-Markovian environment (case 1, dash-dotted red line, $\theta_{A}=\pi /4,\theta_{B}=\theta_{C}=0$) exhibits a similar oscillatory behavior to the fully non-Markovian symmetric case, characterized by an initial rapid decay to sudden death followed by a revival. However, in this configuration, both the intervals of sudden death and the subsequent revival are noticeably smaller, and the overall amplitude of the decay is also increased compared to the symmetric case. This suggests that while the Markovian baths on modes *B* and *C* drive the initial loss, the single non-Markovian bath on mode *A* introduces memory effects that are sufficient to induce revivals, though with diminished strength and duration. The configuration with two photons in a non-Markovian environment (case 2, long-dashed black line, $\theta_{A}=\theta_{B}=\pi /4,\theta_{C}=0$) displays the same qualitative oscillatory pattern observed in the previous cases. However, compared to case 1, the intervals of sudden death are further reduced, and the revival occurs more rapidly, with the steering maintaining a higher overall value (∼0.07 at t=40 ps). This indicates a cumulative effect; coupling more modes to non-Markovian environments enhances the memory-driven recovery process, leading to greater overall robustness of the steering shared by *A* over BC. An intriguing anomaly is observed for the configuration $\theta_{A}=0,\theta_{B}=\pi /4,\theta_{C}=0$ (spaced dashed orange line). Despite only one photon being in a non-Markovian environment (mode *B*), the steering $S^{A-BC}$ exhibits an oscillatory decay that is more rapid than in the fully Markovian case, notably characterized by the absence of complete sudden death over short intervals, unlike the distinct death–revival cycles seen in previous non-Markovian configurations. This counter-intuitive result suggests that the distribution of non-Markovian resources is critical. If the steering party (*A*) is in a Markovian environment, placing a non-Markovian bath on a steered party (*B*) does not aid *A*’s ability to steer and may, through back-action effects mediated by the global tripartite correlations, even accelerate the loss of its steering capability while simultaneously preventing the complete suppression of steerability.

Panel (b) shows the steering from the composite system AB to party *C*. The dynamics here are qualitatively different from panel (a), with the initial values being notably smaller across all configurations compared to the $S^{AB-C}$ case. The fully Markovian symmetric case (dashed blue line, $\theta_{A}=\theta_{B}=\theta_{C}=0$) exhibits a monotonic decay, with steering slowly decreasing and undergoing sudden death by approximately t=40 ps, after which it remains zero with no further revival. In stark contrast, the fully non-Markovian symmetric case (solid green line, $\theta_{A}=\theta_{B}=\theta_{C}=\pi /4$) displays a pronounced oscillatory behavior. After an initial rapid decay leading to a sudden death around t≈9 ps, the steering experiences a strong revival, passing through a maximum value of approximately 0.01 at t≈38 ps. This oscillatory behavior is a clear signature of strong system-environment memory effects, where information lost to the environment is subsequently fed back into the system, temporarily restoring quantum correlations. The behavior of the mixed cases is also highly distinct. The configuration with one non-Markovian photon on the steering side (case 1, dash-dotted red line, $\theta_{A}=\pi /4,\theta_{B}=\theta_{C}=0$) exhibits the same qualitative oscillatory behavior observed in the fully non-Markovian symmetric case, characterized by an initial decay to sudden death followed by a revival. However, in this configuration, the interval of sudden death is notably smaller (occurring around t=10 ps), and the amplitude of the subsequent revival is sufficiently greater than in the symmetric case. This enhanced revival amplitude, despite the shorter death interval, suggests that the asymmetric distribution of non-Markovian resources—where only the steering party *A* benefits from memory effects—can, under certain conditions, amplify the backflow of information and temporarily strengthen the steering capability of AB over *C*, even though the steered party *C* itself is in a Markovian environment. Most notably, the configuration with two non-Markovian photons on the steering side (case 2, long-dashed black line, $\theta_{A}=\theta_{B}=\pi /4,\theta_{C}=0$) exhibits a dramatically different dynamic compared to the previous cases. While it follows the same qualitative pattern of oscillatory behavior, the initial decay is extremely rapid, leading to sudden death after a very short time (t≈5 ps). However, unlike case 1 where a revival with greater amplitude followed, here the steering shows no evidence of recovery; its value drops below the resolution of the plot and remains effectively zero. This suggests that while non-Markovian environments on the steering parties (*A* and *B*) preserve their individual steering over *C* (as seen in panel a), they completely and irreversibly disrupt the collective steering capability of the pair AB over *C*, effectively suppressing the oscillatory recovery observed when only one steering party was non-Markovian. The anomalous case from panel (b) ($\theta_{A}=0,\theta_{B}=\pi /4,\theta_{C}=0$, spaced dashed orange line) here exhibits an oscillatory decay, but notably, it does not undergo sudden death within the simulated time frame, maintaining small oscillations without ever reaching zero. This reinforces that a non-Markovian environment on a non-steering party (*B*) introduces memory effects that induce oscillatory behavior in the steering dynamics of AB→C, yet without causing the complete death of steering observed in other configurations. In summary, the dynamics of tripartite steering in a dephasing W-state are highly sensitive to both the number of photons coupled to non-Markovian environments and the specific partition of the system being considered. For the $S^{A-BC}$ steering measure, non-Markovian effects can induce oscillatory behavior characterized by death–revival cycles, with the intervals of sudden death and revival amplitudes depending critically on how many photons share the memory effects. However, when the steering party itself is Markovian, placing a non-Markovian environment on a steered party leads to oscillatory decay without complete sudden death, yet accelerates the overall loss of steering capability. For the $S^{AB-C}$ steering measure, non-Markovian environments can produce revivals with significantly enhanced amplitudes when asymmetrically distributed, but can also completely suppress oscillatory recovery when both steering parties are non-Markovian, leading to irreversible sudden death. These results highlight the complex interplay between multipartite quantum correlations and environmental memory, demonstrating that non-Markovianity can be a resource for protecting specific types of quantum steering, but its effects are highly configuration-dependent, sometimes enhancing correlations and other times proving detrimental depending on the correlation structure and the distribution of memory effects across the system.

**Figure 4:**
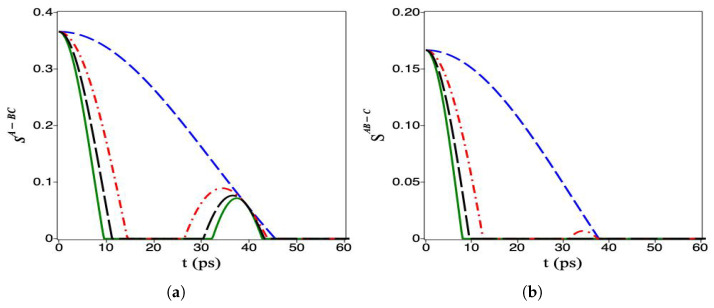
Time evolution of the steering measures $S^{A\to BC}$ (**a**) and $S^{AB\to C}$ (**b**) for a tripartite photonic system initially prepared in a GHZ state (a=1), under Markovian and non-Markovian dephasing dynamics. The steering dynamics are shown for different distributions of environmental memory effects characterized by $\left(\theta_{A},\theta_{B},\theta_{C}\right)$. The system parameters are Δn=0.01, $\nu_{1}=2.676$ PHz, $\nu_{2}=2.692$ PHz, and σ=1.8 THz. Line colors denote different configurations: blue—fully Markovian case (0,0,0); green—fully non-Markovian case (π/4,π/4,π/4); red—single-photon non-Markovian case (π/4,0,0); black—two-photon non-Markovian case (π/4,π/4,0).

The dynamics of quantum steering for a three-mode photonic system, initially prepared in a GHZ-state with parameter a=1 and subject to dephasing, are presented in Figure 4. The figure illustrates the time evolution of two distinct steering measures: $S^{A-BC}$ (panel a), which quantifies the ability of party *A* to steer the composite subsystem BC, and $S^{AB-C}$ (panel b), which quantifies the ability of the composite system AB to steer party *C*. The system evolves under various configurations of Markovian and non-Markovian dephasing environments, characterized by the angle parameter θ, with the spectral properties defined by the detuning Δn=0.01, central frequencies $\nu_{1}=2.676$ PHz (∼704.5 nm), $\nu_{2}=2.692$ PHz (∼700.3 nm), and bandwidth σ=1.8 THz [21].

Panel (a) depicts the steering from mode *A* to the combined modes *B* and *C* for the GHZ-state. The dynamics reveal a strong sensitivity to the environmental configuration, with notable differences compared to the W-state case, particularly in the initial values and decay patterns. The fully Markovian symmetric case (dashed blue line, $\theta_{A}=\theta_{B}=\theta_{C}=0$) exhibits a rapid monotonic decay, with steering undergoing sudden death around t≈45 ps and remaining zero thereafter. This behavior is characteristic of memoryless environments where information is irreversibly lost. In stark contrast, the fully non-Markovian symmetric case (solid green line, $\theta_{A}=\theta_{B}=\theta_{C}=\pi /4$) displays a distinct oscillatory behavior. After an initial decay, the steering experiences a revival, passing through a small maximum around t≈38 ps, followed by another decay. Notably, after t≈42 ps, which the steering ultimately undergoes complete sudden death. This oscillatory behavior, characterized by multiple death–revival cycles, is a direct consequence of the environment’s memory, which allows for a recurrent backflow of information to the system, temporarily restoring quantum correlations before their eventual irreversible loss. The configuration with one non-Markovian photon (case 1, dash-dotted red line, $\theta_{A}=\pi /4,\theta_{B}=\theta_{C}=0$) exhibits a behavior qualitatively similar to the fully non-Markovian symmetric case, with oscillatory decay and revival. However, in this configuration, the amplitude of the revival is notably larger, and the steering maintains a higher value throughout the dynamics compared to the symmetric non-Markovian case. This suggests that when only the steering party *A* benefits from memory effects, the backflow of information is more efficiently channeled into preserving its steering capability over BC, despite modes *B* and *C* being in Markovian environments. The configuration with two non-Markovian photons on the non-steering sides (case 2, long-dashed black line, $\theta_{A}=\theta_{B}=\pi /4,\theta_{C}=0$) displays yet another distinct dynamic. It follows the same oscillatory pattern but with a much longer-lived revival. After an initial decay to near-zero around t≈30 ps, the steering experiences a narrow revival that persists with small oscillations up to t≈44 ps before eventually decaying. This indicates a synergistic effect; coupling more modes to non-Markovian environments enhances the memory-driven recovery process, leading to shorted preservation of the steering shared by *A* over BC.

Panel (b) shows the steering from the composite system AB to party *C* for the GHZ-state. The dynamics here are qualitatively different from panel (a), with overall smaller initial values and distinct decay patterns across configurations. The fully Markovian symmetric case (dashed blue line, $\theta_{A}=\theta_{B}=\theta_{C}=0$) exhibits a monotonic decay, with steering undergoing sudden death around t≈38 ps and remaining zero thereafter with no revival. The fully non-Markovian symmetric case (solid green line, $\theta_{A}=\theta_{B}=\theta_{C}=\pi /4$) displays a markedly different behavior. After an initial rapid decay, the steering undergoes sudden death around t≈8 ps and remains zero thereafter, exhibiting no revival. This absence of recovery indicates that, despite the non-Markovian character of the environments, the collective steering capability of AB over *C* is irreversibly lost. The configuration with one non-Markovian photon on the steering side (case 1, dash-dotted red line, $\theta_{A}=\pi /4,\theta_{B}=\theta_{C}=0$) exhibits a similar oscillatory pattern to the fully non-Markovian symmetric case, but with notably different characteristics. The initial decay is slower, and the revival amplitude is smaller, with steering maintaining a smaller value throughout the dynamics. However, like the symmetric case, it undergoes sudden death around t≈38 ps with no further revival. This suggests that while a single non-Markovian environment on the steering party *A* enhances the steering capability, it cannot prevent the eventual sudden death when the steered party *C* is in a Markovian environment. Most notably, the configuration with two non-Markovian photons on the steering side (case 2, long-dashed black line, $\theta_{A}=\theta_{B}=\pi /4,\theta_{C}=0$) exhibits a dramatically different dynamic. It decays monotonically without any oscillatory behavior, undergoing sudden death around t≈10 ps and remaining zero thereafter. This suggests that while non-Markovian environments on the steering parties (*A* and *B*) preserve their individual steering over *C* (as seen in panel a), they completely disrupt the oscillatory recovery of the collective steering capability of the pair AB over *C*, leading to irreversible sudden death.

In summary, the dynamics of steering correlations in a dephasing GHZ-state are highly sensitive to both the number of photons coupled to non-Markovian environments and the specific partition of the system being considered. For the $S^{A-BC}$ steering measure, non-Markovian effects can induce oscillatory behavior with multiple death–revival cycles, and in some configurations, lead to prolonged preservation of steering without complete sudden death. In contrast, for the $S^{AB-C}$ steering measure, non-Markovian environments on the steering parties can either induce a single revival before irreversible sudden death or, in the case of two non-Markovian steering parties, result in rapid decay with no revival whatsoever. These results highlight that the efficacy of non-Markovian environments depends critically on which subsystems they are coupled to, relative to the steering and steered parties. The complex interplay between multipartite quantum correlations and environmental memory demonstrates that non-Markovianity can serve as a resource for protecting specific types of quantum steering in GHZ-states, but its effects are highly configuration-dependent, sometimes leading to enhanced preservation and other times resulting in accelerated irreversible loss. These results indicate that environmental memory effects can substantially modify the decay dynamics of multipartite steering by enabling partial recovery of coherence through information backflow mechanisms.

Furthermore, we conclude with a remark on the comparison between Markovian and non-Markovian dephasing regimes.

The results presented above demonstrate clear qualitative differences between Markovian and non-Markovian dephasing dynamics in multipartite photonic systems. In the Markovian regime, the steering measures generally exhibit monotonic decay due to the irreversible loss of coherence into the environment. By contrast, the non-Markovian regime is characterized by oscillatory and nonmonotonic behavior associated with environmental memory effects and partial information backflow from the reservoir to the system.

Importantly, non-Markovianity does not necessarily imply an enhancement or preservation of multipartite quantum steering. In the present model, we observe that non-Markovian dephasing can, in several parameter regimes, induce a stronger suppression of steering compared with the Markovian limit, despite the presence of revival structures. This indicates that revival phenomena alone cannot be interpreted as a signature of increased robustness of quantum correlations.

This behavior originates from the competition between memory-induced information backflow and phase-destroying processes acting on the off-diagonal elements of the density matrix, which encode multipartite coherence. Depending on the bipartition and the distribution of environmental memory across the subsystems, this interplay may either transiently restore steering correlations or accelerate their overall degradation. These observations highlight the strongly configuration-dependent role of environmental memory in multipartite quantum systems.

## 7. Conclusions

In this work, we have systematically investigated the non-Markovian dynamics of quantum steering in a tripartite photonic system, with a particular focus on how environmental memory effects influence the temporal evolution of quantum correlations. We developed a comprehensive theoretical framework beginning with the single-photon dephasing model, which we extended to describe three independent photons interacting with their respective environments. By employing a matrix representation in the polarization basis, we were able to accurately model the dephasing mechanisms affecting each photon and subsequently derive the dynamics for composite three-photon systems. We applied this framework to two distinct classes of initial states: the W-type entangled state and the GHZ-type mixed entangled state, examining their evolution under both Markovian and non-Markovian dephasing dynamics. The steering measures $S^{A-BC}$ and $S^{AB-C}$ were used to quantify the directional quantum correlations between different bipartitions of the tripartite system, providing insight into how the steering is distributed and preserved throughout the network. Our results reveal that the dynamics of quantum steering are highly sensitive to both the number of photons coupled to non-Markovian environments and the specific partition of the system being considered. For the W-state, the $S^{A-BC}$ steering measure exhibited oscillatory behavior characterized by death–revival cycles, with the intervals of sudden death and revival amplitudes depending critically on how many photons shared the memory effects. Notably, when the steering party itself was Markovian, placing a non-Markovian environment on a steered party led to oscillatory decay without complete sudden death, yet paradoxically accelerated the overall loss of steering capability. For the $S^{AB-C}$ steering measure in W-states, non-Markovian environments produced revivals with significantly enhanced amplitudes when asymmetrically distributed, but completely suppressed oscillatory recovery when both steering parties were non-Markovian, leading to irreversible sudden death. For the GHZ-state, the dynamics displayed even richer behavior. In the $S^{A-BC}$ case, non-Markovian effects induced multiple death–revival cycles, with some configurations leading to prolonged preservation of steering without complete sudden death. The asymmetric distribution of memory effects proved particularly beneficial when only the steering party benefited from non-Markovian environment, resulting in enhanced revival amplitudes. In contrast, the $S^{AB-C}$ steering measure for GHZ-states showed that non-Markovian environments on the steering parties could either induce a single revival before irreversible sudden death or, in the case of two non-Markovian steering parties, result in rapid decay with no revival whatsoever. These findings demonstrate that the efficacy of non-Markovian environments depends critically on which subsystems they are coupled to, relative to the steering and steered parties.

Importantly, our analysis shows that non-Markovianity does not universally enhance or preserve multipartite quantum steering. Depending on the configuration of the system and the distribution of memory effects among the steering and steered subsystems, environmental memory may either induce temporary revivals through information backflow or accelerate the degradation of quantum correlations. In the present dephasing model, several non-Markovian configurations were found to produce stronger damping of steering than their Markovian counterparts, despite the appearance of oscillatory revival dynamics. This behavior highlights the subtle interplay between coherence exchange, phase accumulation, and environmental memory in multipartite photonic systems. The stronger suppression observed in certain non-Markovian regimes can be attributed to the competition between information backflow and destructive interference effects in the off-diagonal coherence terms responsible for steering. Although memory effects may temporarily restore coherence, they can also enhance oscillatory decoherence processes, leading to faster overall decay depending on the considered bipartition and initial state. Therefore, the role of non-Markovianity should be regarded as highly configuration-dependent rather than universally beneficial. Our results contribute to the fundamental understanding of open quantum systems and provide practical insights for quantum information processing tasks that rely on the preservation of steering-based quantum correlations. The present work is primarily focused on an analytically tractable non-Markovian dephasing model that enables a clear characterization of multipartite steering dynamics. More realistic environments, such as Ohmic, sub-Ohmic, super-Ohmic, Drude–Lorentz, or structured spectral-density reservoirs, may lead to richer dynamical features and constitute an important direction for future investigations. Such extensions would generally require fully numerical treatments beyond the scope of the current analytical framework. Nevertheless, the ability to engineer environmental memory effects remains a promising route to control quantum correlations in photonic networks. Understanding how different reservoir configurations influence steering robustness may provide useful guidelines for the implementation of quantum communication, quantum cryptography, and distributed quantum-information protocols in realistic noisy environments. Future work may explore the extension of these results to larger photonic networks, the influence of different spectral densities, and the optimization of environmental parameters to maximize the preservation of quantum correlations in realistic experimental settings.

## Data Availability

The original contributions presented in this study are included in the article. Further inquiries can be directed to the corresponding author.

## Appendix A. Explicit Steering Matrices

In this Appendix, we provide the explicit matrix representations of the steering witnesses $\tau^{1}$ and $\tau^{2}$ introduced in the main text. These expressions are omitted from the main manuscript for readability, as they involve large 8×8 matrices whose structure is fully determined by the reduced density matrix elements and the local dephasing functions $\eta_{A}\left(t\right)$, $\eta_{B}\left(t\right)$, and $\eta_{C}\left(t\right)$. The matrices are presented here for completeness and to allow direct verification of the steering criteria based on their eigenvalue spectra.

1. W-state steering matrices:
  The explicit form of $\tau_{ABC}^{1(W)}$ is
  (A1) $$\tau_{ABC}^{1(W)}=\frac{1}{3}\left(\begin{array}{cccccccc} \alpha & 0 & 0 & 0 & 0 & 0 & 0 & 0\\ 0 & \frac{\sqrt{3}}{3}+\alpha & \left(\frac{\sqrt{3}}{3}+\alpha \right)\eta_{B}\eta_{C}^{*} & 0 & \frac{\sqrt{3}}{3}\eta_{A}\eta_{C}^{*} & 0 & 0 & 0\\ 0 & \left(\frac{\sqrt{3}}{3}+\alpha \right)\eta_{B}^{*}\eta_{C} & \frac{\sqrt{3}}{3}+\alpha & 0 & \frac{\sqrt{3}}{3}\eta_{A}\eta_{B}^{*} & 0 & 0 & 0\\ 0 & 0 & 0 & 0 & 0 & 0 & 0 & 0\\ 0 & \frac{\sqrt{3}}{3}\eta_{A}^{*}\eta_{C} & \frac{\sqrt{3}}{3}\eta_{A}^{*}\eta_{B} & 0 & \frac{\sqrt{3}}{3}+\alpha & 0 & 0 & 0\\ 0 & 0 & 0 & 0 & 0 & \alpha & \alpha \;\eta_{B}\eta_{C}^{*} & 0\\ 0 & 0 & 0 & 0 & 0 & \alpha \;\eta_{B}^{*}\eta_{C} & \alpha & 0\\ 0 & 0 & 0 & 0 & 0 & 0 & 0 & 0 \end{array}\right).$$
  In this representation, the global prefactor 1/3 ensures the proper normalization of the density matrix and reflects the statistical weight inherited from the underlying W-state construction. The matrix elements inside the brackets therefore represent dimensionless population and coherence weights. These weights naturally separate into distinct physically meaningful sectors: The coefficient α characterizes the weakest population sector and is associated with mixing-induced or noise-like contributions introduced by the state construction. The constant contribution $\sqrt{3}/3$ represents the intrinsic population and coherence scale originating from the ideal W-state component. The combined coefficient $\left(\sqrt{3}/3+\alpha \right)$ governs the dominant population sector and also appears in several coherence terms, indicating that both the intrinsic W-state structure and the mixing parameter α jointly determine the strength of quantum correlations between basis states.
  Similarly, $\tau_{ABC}^{2(W)}$ is
  (A2) $$\tau_{ABC}^{2(W)}=\frac{1}{18}\left(\begin{array}{cccccccc} 2 & 0 & 0 & 0 & 0 & 0 & 0 & 0\\ 0 & 3 & 2\;\eta_{B}\eta_{C}^{*} & 0 & 2\;\eta_{A}\eta_{C}^{*} & 0 & 0 & 0\\ 0 & 2\;\eta_{B}^{*}\eta_{C} & 4 & 0 & 2\;\eta_{A}\eta_{B}^{*} & 0 & 0 & 0\\ 0 & 0 & 0 & 1 & 0 & 0 & 0 & 0\\ 0 & 2\;\eta_{A}^{*}\eta_{C} & 2\;\eta_{A}^{*}\eta_{B} & 0 & 4 & 0 & 0 & 0\\ 0 & 0 & 0 & 0 & 0 & 1 & 0 & 0\\ 0 & 0 & 0 & 0 & 0 & 0 & 2 & 0\\ 0 & 0 & 0 & 0 & 0 & 0 & 0 & 1 \end{array}\right).$$
  This expression is derived for the W state under local non-Markovian dephasing in the photonic system, where the off-diagonal elements are damped by products of the dephasing functions $\eta_{A}\left(t\right)$, $\eta_{B}\left(t\right)$, and $\eta_{C}\left(t\right)$ corresponding to the qubits that differ between basis states. Due to the mathematical equivalence of the effective density operators derived in the non-Markovian dephasing model to those arising in photonic systems where one photon experiences asymmetric decoherence, the steering quantifiers $S^{A\to BC}$ and $S^{AB\to C}$ exhibit behaviors modulated by the environmental memory effects.
2. GHZ-state steering matrices: The matrices $\tau_{ABC}^{1(G)}$ and $\tau_{ABC}^{2(G)}$ are given by:
  (A3) $$\tau_{ABC}^{1(G)}=\frac{1}{3}\left(\begin{array}{cccccccc} K_{c} & 0 & 0 & 0 & 0 & 0 & 0 & \frac{\sqrt{3}}{2}a\;\eta_{A}\eta_{B}\eta_{C}\\ 0 & K_{d}^{\left(1\right)} & 0 & 0 & 0 & 0 & 0 & 0\\ 0 & 0 & K_{d}^{\left(1\right)} & 0 & 0 & 0 & 0 & 0\\ 0 & 0 & 0 & K_{d}^{\left(2\right)} & 0 & 0 & 0 & 0\\ 0 & 0 & 0 & 0 & K_{d}^{\left(2\right)} & 0 & 0 & 0\\ 0 & 0 & 0 & 0 & 0 & K_{d}^{\left(1\right)} & 0 & 0\\ 0 & 0 & 0 & 0 & 0 & 0 & K_{d}^{\left(1\right)} & 0\\ \frac{\sqrt{3}}{2}a\;\eta_{A}^{*}\eta_{B}^{*}\eta_{C}^{*} & 0 & 0 & 0 & 0 & 0 & 0 & K_{c} \end{array}\right),$$
  where $K_{c}\equiv \sqrt{3}\;c+3\alpha \;\left(c+d\right),\;K_{d}^{\left(1\right)}\equiv \sqrt{3}\;d+6\alpha \;d,\;K_{d}^{\left(2\right)}\equiv \sqrt{3}\;d+3\alpha \;\left(c+d\right)$, and the shorthand $\eta_{A}=\eta_{A}\left(t\right)$, $\eta_{B}=\eta_{B}\left(t\right)$, $\eta_{C}=\eta_{C}\left(t\right)$ (and similarly for complex conjugates) has been used, and $\rho_{BC}\left(t\right)={\mathrm{Tr}}_{A}\left[\rho \left(t\right)\right]$.
  (A4) $$\tau_{ABC}^{2(G)}=\frac{1}{6}\left(\begin{array}{cccccccc} L_{c} & 0 & 0 & 0 & 0 & 0 & 0 & a\;\eta_{A}\eta_{B}\eta_{C}\\ 0 & L_{d} & 0 & 0 & 0 & 0 & 0 & 0\\ 0 & 0 & L_{d} & 0 & 0 & 0 & 0 & 0\\ 0 & 0 & 0 & L_{d} & 0 & 0 & 0 & 0\\ 0 & 0 & 0 & 0 & L_{d} & 0 & 0 & 0\\ 0 & 0 & 0 & 0 & 0 & L_{d} & 0 & 0\\ 0 & 0 & 0 & 0 & 0 & 0 & L_{d} & 0\\ a\;\eta_{A}^{*}\eta_{B}^{*}\eta_{C}^{*} & 0 & 0 & 0 & 0 & 0 & 0 & L_{c} \end{array}\right).$$
  where $L_{c}=3\left(c+d\right),\;L_{d}=5d+c$, and $\rho_{C}\left(t\right)={\mathrm{Tr}}_{AB}\left[\rho \left(t\right)\right]=\mathrm{diag}\left(c+6d,c+6d\right)$ (again up to normalization). The matrices are Hermitian, with off-diagonal coherence terms preserved only in the |000〉↔|111〉 sector and scaled by the respective coefficients in the steering witnesses.
3. Eigenvalue criterion: Steering is certified when the corresponding matrix $\tau^{1}$ or $\tau^{2}$ exhibits at least one negative eigenvalue.
